# An LSPR-Based Kinetic Framework for Polyelectrolyte Molecular Weight Determination: A Proof-of-Concept Study

**DOI:** 10.1021/acs.analchem.5c05449

**Published:** 2026-01-21

**Authors:** Ryan M. Mooney, Charles Brainin, Malkiat S. Johal

**Affiliations:** Department of Chemistry, 2529 Pomona College, 645 N. College Avenue, Claremont, California 91711, United States

## Abstract

Determining the molecular weight of polyelectrolytes remains a persistent challenge because conventional techniques such as size-exclusion chromatography (SEC) often require added salt, calibration against neutral polymers, and complex optimization to overcome chain–column interactions. Here we introduce a kinetics-based method that employs Localized Surface Plasmon Resonance (LSPR) to measure the real-time electrostatic complexation of poly­(ethylenimine) (PEI) and poly­[1-[4-(3-carboxy-4-hydroxyphenylazo)­benzenesulfonamido]-1,2-ethanediyl, sodium salt] (PAZO). By extracting association and dissociation rate constants from concentration-dependent binding transients, we establish a calibration-free approach to determining the number-average molecular weight (*M*_*n*_) of polyelectrolytes under dilute, salt-free conditions. Using the known *M*_*n*_ of PEI, the *M*_*n*_ of PAZO was calculated as 257,400 g mol^–1^, corresponding to a degree of polymerization of 642. This analysis assumes symmetry of the association rate constant (*k*_*on*_) across binding orientations, an assumption justified by the reciprocal nature of electrostatic interactions. The results highlight LSPR-based kinetics as a powerful, surface-sensitive alternative to traditional methods, offering a broadly adaptable strategy for characterizing charged polymers and other biomolecular systems. This study is presented as a proof-of-concept demonstration, emphasizing the novelty and feasibility of the LSPR-based kinetic framework rather than exhaustive validation of molecular weight determinations. By focusing on methodological innovation, the work highlights a generalizable strategy for polyelectrolyte characterization that can be extended and refined in future investigations.

## Graphical abstract

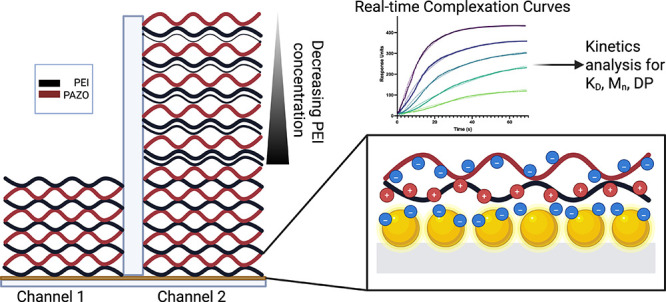

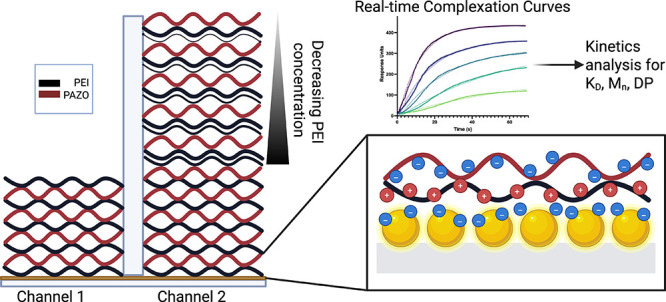

## Introduction

The construction of well-defined polyelectrolyte multilayers (PEMUs) has attracted significant interest due to their broad utility in applications such as biomaterial surface coatings,^1^ drug-delivery platforms,^2^ and biosensors.^3^ Real-time measurements of polyelectrolyte complexation have been explored using gravimetric and optical methods,^4,5^ yet direct observation of PEMU formation dynamics remains limited. Polyelectrolytes also serve as valuable model systems for understanding the behavior of more complex charged biomolecules, including DNA and proteins in aqueous environments.^6^ Various strategies have been employed to assemble PEMUs, including Langmuir–Blodgett deposition,^7^ vapor deposition,^8^ ionic self-assembly,^9^ drop-casting,^10^ and spin-assembly.^4^ Despite these advances, experimental studies capturing the real-time process of PEMU formation and polyelectrolyte complexation are still lacking.

Determining the number-average molecular weight (*M*_*n*_) of polyelectrolytes remains a significant challenge due to the distinctive features of charged polymers. Polyelectrolyte chains carry a high density of charges, which generates long-range electrostatic repulsion and an associated counterion cloud. Even under dilute conditions, these polymers exhibit strong correlations with one another and with their counterions.^11^ At low ionic strength, such as in pure water, intrachain charge repulsion drives the adoption of highly extended conformations.^12^ Conventional techniques for molecular weight characterization developed for neutral polymers—such as osmometry, light scattering, and size exclusion chromatography (SEC)—are therefore difficult to apply directly to polyelectrolytes. In aqueous SEC, extended coils may elute abnormally, or fail to elute altogether, due to pore exclusion or interactions with charged column packing.^13^ To mitigate these effects, high salt concentrations are often employed to induce coil contraction and improve hydrodynamic behavior, thereby enabling approximate size-based separations. However, these conditions require careful optimization and may compromise the intrinsic properties of the polymer. SEC derived *M*_n_ can lead to substantial underestimation of *M*_n_ when applied to polyelectrolytes since added salt is introduced to the solution to increase hydrodynamic behavior. The molar mass derived by SEC in salt-free eluent can thus exceed the molar mass in salt-containing eluent.^19^ When SEC is applied to polyelectrolytes in aqueous solution, there can be severe discrepancies of *M*_n_ resulting from Donnan salt exclusion. When salt-containing solutions are employed in SEC experiments, elution volume decreases and thus determined *M*_n_ is underestimated. Moreover, SEC calibration curves are typically derived from neutral polymers, introducing substantial error when applied to charged systems.^20^ The dependence of polyelectrolyte conformation and elution on multiple solution variables, including salt concentration, pH, and lack of solubility of polyelectrolytes in organic solvents compatible with SEC presents further experimental difficulties.^21^

In this study, dilute, salt-free conditions are chosen to maintain polyelectrolyte chains in a rigid, extended conformation arising from intramolecular electrostatic repulsion. This configuration promotes complexation interactions along the entire polymer backbone while minimizing extraneous variables such as pH and salt concentration. Poly­(ethylenimine) (PEI) serves as the polycation, a well-characterized polymer widely used in surface chemistry as an effective immobilization reagent for anchoring biomolecules in interfacial studies.^3,22^ PEI has a number-average molecular weight of ∼25,000 g mol^–1^. The polyanion poly­[1-[4-(3-carboxy-4-hydroxyphenylazo)­benzenesulfonamido]-1,2-ethanediyl, sodium salt] (PAZO) is reported to have a molecular weight in the range of 65,000–100,000 g mol^–1^, but no cited *M*_*n*_. PAZO was chosen as the polyelectrolyte to determine *M*_*n*_ using this method given its lack of solubility in the most common solvents used for SEC experiments and its undetermined *M*_*n*_.

Here we address this gap by demonstrating a kinetics-based approach that leverages real-time binding interactions between PAZO and PEI under salt-free conditions to extract *M*_*n*_ and the degree of polymerization (DP). By moving beyond hydrodynamic size-based methods and exploiting electrostatic complexation kinetics, this study provides a calibration-free strategy for characterizing polyelectrolytes under conditions where traditional methods fail.

Localized Surface Plasmon Resonance (LSPR) is a label-free biosensing technique capable of monitoring a wide range of molecular interactions, including protein–protein binding.^23,24^ The instrument employs a dual-channel design in which one channel functions as the active binding surface and the other as a nonbinding reference. Binding events are detected through shifts in the localized plasmon resonance wavelength at the gold nanoparticle surface, and the difference between the two channels ensures that only true binding signals are recorded. LSPR monitors changes in the refractive index near the gold nanoparticle surface when an analyte associates with an immobilized receptor, enabling detection of both low- and high-affinity interactions that are often inaccessible by other methods.^25^ In LSPR, changes in the local refractive index are detected as shifts in the nanoparticle extinction peak wavelength (Δλ). In this work, the instrument reports these changes in response units (RU), which are linearly proportional to the LSPR wavelength shift and therefore to the surface mass concentration of bound analyte. 1000 RU corresponds to ∼100 pg of bound mass at the sensor surface and the wavelength resolution of LSPR is 0.1 pm.

As a surface-sensitive technique, LSPR provides a powerful and complementary approach for probing polyelectrolyte behavior by allowing real-time monitoring of complexation between oppositely charged polyions. Unlike conventional methods that rely on indirect chain properties, LSPR directly quantifies intermolecular binding kinetics, offering access to parameters that are otherwise difficult to measure (see Table 1 for examples of common binding systems studied by LSPR and related instrumentation). By correlating the real-time complexation kinetics of PEI and PAZO with known binding constants, we demonstrate a calibration-free method for estimating the number-average molecular weight of polyelectrolytes (PAZO in this study) under dilute, salt-free conditions. This LSPR-based strategy establishes a versatile platform for characterizing charged polymers and can be readily extended to other synthetic and biomolecular systems.

**Table 1:** Representative Applications of LSPR in Kinetic Characterization

System Type	Representative Study	System Analyzed	Technique	Relevance
Protein–protein	Bhagawati M, You C, Piehler J (2013) *Anal. Chem*.^14^	His-tagged protein–protein interaction	LSPR	Measured real-time binding kinetics (*k*_*on*_, *k*_*off*_) for a reversible protein complex. Demonstrated LSPR imaging with ∼30 protein molecules detectable in a diffraction-limited area.
Protein-Small Molecule	Haes AJ, Van Duyne RP (2002) *J. Am. Chem. Soc*.^15^	Biotin–Streptavidin	LSPR	Obtained equilibrium binding curves and affinity of the biotin-Streptavidin interaction with LSPR. LSPR spectral shifts enabled label-free detection down to low-picomolar/high-femtomolar analyte levels.
Protein–DNA	Guo L, Kim D-H (2012) *Biosensors & Bioelectronics* ^16^	Thrombin binding to a DNA aptamer (surface-tethered) with antibody sandwich for signal amplification	LSPR	Real-time LSPR shift observed upon aptamer–thrombin binding, and a secondary antithrombin antibody step amplified the signal.
Biomarker SPRi immunoassay	Hendriks et al., (2022) *Anal. Biochem*.^17^	SPR imaging (SPRi) chip with arrayed capture probes for disease biomarkers	SPRi	Developed a kinetic method using a biphasic binding-sites model to fit SPRi binding curves.
Polymer–Polymer	Ghostine RA, Markarian MZ, Schlenoff JB (2013) *J. Am. Chem. Soc*.^18^	Layer-by-layer assembly of PDADMAC (polycation) and PSS (polyanion) films	QCM-D	Tracked multilayer build-up kinetics and charge uptake. Found that polycation adsorption causes surface charge overcompensation.

The present work is intended as a proof-of-concept, establishing the feasibility of applying LSPR-based kinetic analysis for polyelectrolyte molecular weight determination. Rather than providing a comprehensive validation across systems, our goal here is to introduce the methodological framework and illustrate its potential as a broadly adaptable alternative to conventional techniques.

## Experimental Section

### Materials

PEI (*M* *_W_* ≈ 25 000 g mol^–1^, CAS 9002–98–6; *M*_*n*_ ≈ 10,000 g mol^–1^, mixture of linear and branched; *M*_*o*_ ≈ 43.07 g mol^–1^) and PAZO (*M*_*W*_ ≈ 65 000–100 000 g mol^–1^, CAS 219957–04–7, *M*_*o*_ ≈ 401 g mol^–1^), were obtained from Sigma-Aldrich. Aqueous solutions of polyelectrolytes were prepared in ultrapure water (resistivity >18MΩ cm, Milli-Q) at concentrations determined by the monomeric mass of 1 mM, 0.5 mM, 025 mM, 0.0125 mM, and 0.0625 mM. Localized Surface Plasmon Resonance (LSPR) data was collected on Nicoya OpenSPR-XT using High-Capacity Carboxyl Sensors (SEN-HS-8-COOH, Nicoya) and Nicoya’s cool incident light (LED-COOL, Nicoya) following the supplier’s carboxyl sensor guide protocol.

### Experimental Protocols

Carboxyl sensors were rinsed in ultrapure water for 15 s on each side of the sensor and dried in a stream of N_2_ gas, and 80% isopropanol was used to clean the flow cell surface and allowed to dry prior to docking. Milli-Q was used as the running buffer for all experiments. The high-capacity carboxyl sensor was first cleaned using a 5 mM hydrochloric acid injection. Five bilayers, starting with PEI deposition onto the negatively charged carboxylate-functionalized surface, were self-assembled on both channels. Five bilayers were built prior to kinetic analysis because it has been shown that bilayers of the same concentration build up linearly after three bilayers.^16^ On the active channel, 1 mM PAZO was injected until complete charge reversal. Charge-reversal of the active channel permits subsequent PEI binding, whereas binding to the reference channel is blocked due to electrostatic repulsion. This allows for the reference channel to correct for bulk-shift of chromophore-containing polyelectrolytes and ensures only biding and complexation interactions on the surface are recorded. The 0.5 mM PEI solution then was injected over both channels, where complexation is seen in real-time uniquely to the PAZO surface. To ensure equal thickness of bilayers, a 1 mM PEI solution was then injected to correct for decreased complexation given the lower concentration of the analyte. To regenerate the active channel surface, a 1 mM PAZO ligand was flown on the active channel until complete charge reversal. This procedure was repeated for the 0.25 mM, 0.0125 mM and 0.0625 mM solutions of PEI. This entire procedure, starting with the buildup of five 1 mM bilayers was then repeated using PAZO as the analyte and PEI as the ligand. Experiments for each polyelectrolyte gradient were assayed in duplicate.

### Data Analysis

The electrostatic complexation between poly­(ethylenimine) (PEI, denoted P) and PAZO (denoted Pa) can be described by the bimolecular association reaction shown in eq 1:

1 $$P+\mathrm{Pa}\overset{k_{on}}{\underset{k_{off}}{⇌}}P\text{−}\mathrm{Pa}$$

The rate law corresponding to eq 1 can be written as

2 $$\frac{d\left[P\text{−}\mathrm{Pa}\right]}{dt}=k_{on}\left[P\right]\left[\mathrm{Pa}\right]-k_{off}\left[P\text{−}\mathrm{Pa}\right]$$

Applying pseudo-first-order conditions, where the concentration of PEI remains effectively constant ([P] ≈ [P]_0_) because it is continuously flowed over the PAZO surface at a fixed concentration, the rate law simplifies to

3 $$\frac{d\left[P\text{−}\mathrm{Pa}\right]}{dt}=k_{on}{\left[P\right]}_{0}\left[\mathrm{Pa}\right]-k_{off}\left[P\text{−}\mathrm{Pa}\right]$$

The integrated solution of this rate law yields

4 $$\left[P\text{−}\mathrm{Pa}\right]\left(t\right)={\left[P\right]}_{0}\frac{k_{on}{\left[\mathrm{Pa}\right]}_{0}}{k_{on}{\left[\mathrm{Pa}\right]}_{0}+k_{off}}\left(1-e^{-(k_{on}{\left[P\right]}_{0}+k_{off})t}\right)$$

This expression describes the time-dependent formation of the PEI–PAZO complex, where the exponential term captures the approach to equilibrium. By grouping constants, the relationship can be expressed more simply as

5 $$\left[P\text{−}\mathrm{Pa}\right]\left(t\right)=C\left(1-e^{-k_{obs}t}\right)$$

By fitting the kinetic transients to association curves obtained from PEI adsorption onto immobilized PAZO, the observed rate constants (*k*_*obs*_) can be extracted for various concentrations of PEI ([P]_0_). The relationship between *k*_*obs*_ and [P]_0_ is described by

6 $$k_{obs}=k_{on}{\left[P\right]}_{0}=k_{eff}$$

A plot of *k*_*obs*_ versus [P]_0_ yields *k*_*on*_ as the slope and *k*_*off*_ as the *y*-intercept. From these values, the equilibrium binding constant *K*_*D*_ can be calculated as

7 $$K_{D}=\frac{k_{off}}{k_{on}}$$

Values of *K*_*D*_ were determined for both PEI complexation onto a PAZO-coated surface and PAZO complexation onto a PEI-coated surface. The same equations apply in both cases; for the PAZO-on-PEI analysis, the roles of [Pa] and [P] are simply exchanged.

All curve fitting, nonlinear regression, and statistical analyses were performed using GraphPad Prism 10 (GraphPad Software, San Diego, CA), and figures were compiled using Biorender.

## Results and Discussion

The electrostatic interactions between PEI and PAZO were monitored in real time using LSPR. In this technique, binding events are detected as shifts in Response Units (RU), which are proportional to the mass of material adsorbed onto the carboxyl-functionalized gold sensor surface and, by extension, to the number of molecules bound.

PEI self-assembles onto the negatively charged, carboxylate-functionalized gold nanoparticles. Complete charge reversal after PEI deposition then allows self-assemble of negatively charged PAZO. The charge reversal mechanism after each deposition of polyelectrolyte enables selective adsorption. Figure 1 illustrates the linear buildup of five PEMUs, each bilayer formed by sequential deposition of 1 mM PEI followed by 1 mM PAZO, with solutions flowed until steady-state equilibrium was reached. RU values recorded after each deposition step show a clear, stepwise increase in surface mass. The linearity observed in the first five bilayers is consistent with previous literature reports on PEMU formation^3,4^ and validates the use of LSPR for capturing the layer-by-layer self-assembly process of PEMUs in real time. This confirmation provides the foundation for applying LSPR not only as a qualitative probe of multilayer buildup but also as a quantitative tool for kinetic analysis of polyelectrolyte complexation.

**Figure 1:**
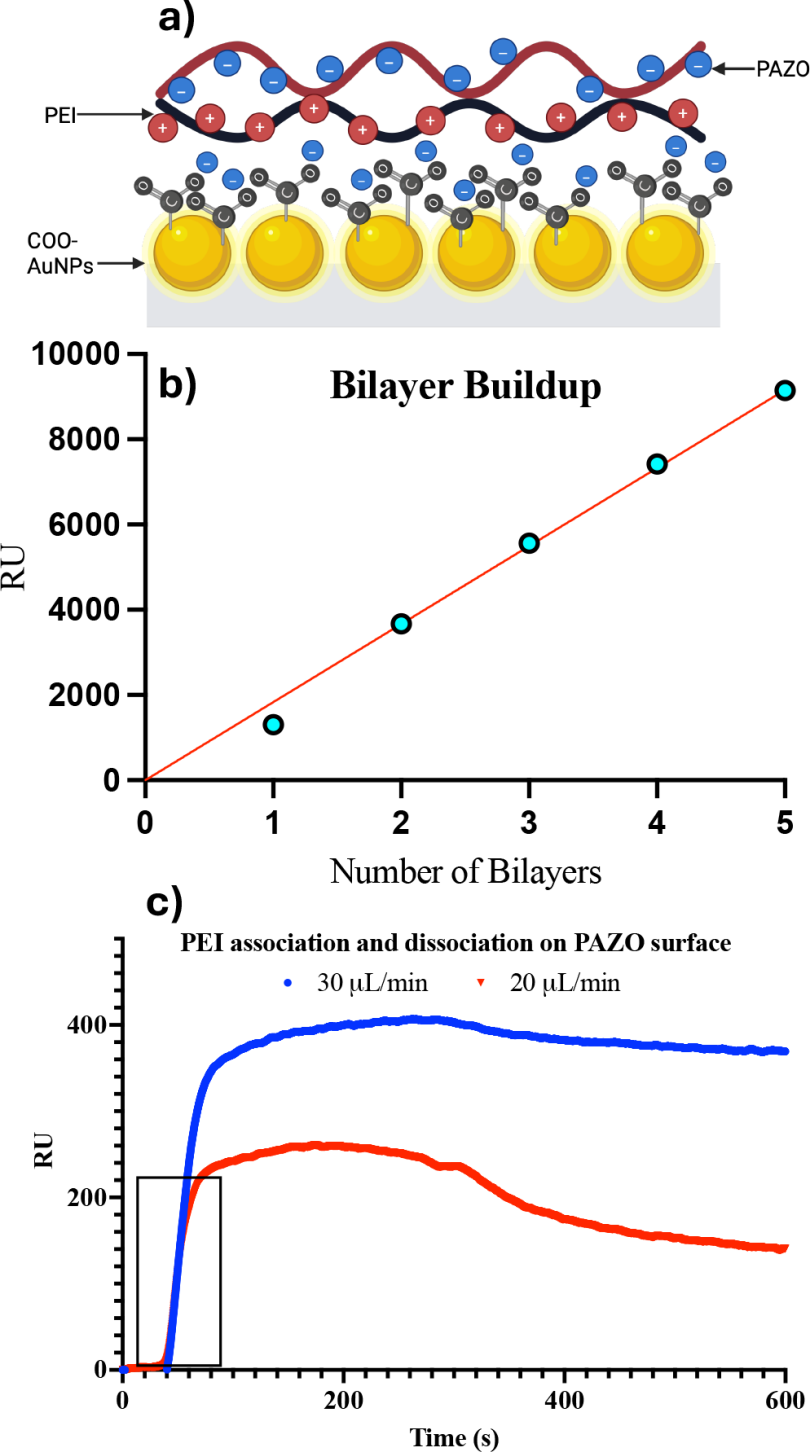
a) LSPR sensor schematic with carboxylate functionalized gold nanoparticles and one PEMU. b) Linear buildup of five polyelectrolyte bilayers recorded by LSPR. Each bilayer was formed by sequential deposition of 1 mM PEI followed by 1 mM PAZO onto the sensor surface. Response Units (RU) were recorded after each deposition step once steady-state equilibrium was reached. A simple linear regression fit of RU versus bilayer number shows agreement with a coefficient of determination *R* ^2^ = 0.9922, confirming that multilayer growth is uniform and consistent with the expected stepwise assembly of polyelectrolyte multilayers. c) Flow rate study of PEI deposition on PAZO. At time = 0 s, 1 mM PEI began flowing into the flow cell at the indicated flow rate. The box indicates the initial association phase most sensitive to *k*_obs_ determination. Dissociation phase begins at time = 300 s.

To evaluate whether the deposition kinetics were influenced by mass-transport limitations, we performed flow-rate–dependence experiments using PEI (1 mM) injected over a PAZO (1 mM) surface at 20 and 30 μL/min (Figure 1c). Although the lower flow rate produced a reduction in RU_max_, the shape and time dependence of the association phase were identical across both conditions. Specifically, the initial rise in signal during the first 20–40 s, the region most sensitive to diffusion-controlled behavior, showed indistinguishable slopes and curvature at both flow rates, indicating that analyte transport to the surface remained sufficiently rapid and that the system was predominantly reaction-controlled.

The absence of a broadened or delayed association front at 20 μL/min, together with the near-superimposable exponential approach to steady-state, confirms that diffusion-layer depletion effects were minimal under our experimental conditions. The difference in RU_max_ observed at lower flow likely reflects minor alterations in boundary-layer thickness rather than a change in intrinsic binding kinetics. Importantly, all equilibrium and kinetic measurements used in following experiments were conducted at the higher flow rate (30 μL/min), where mass-transport contributions are further reduced. Collectively, these control experiments demonstrate that under a constant flow of 30 μL/min, the deposition process operates in a regime where diffusion-controlled contributions are minimal, validating the use of a reaction-limited kinetic model for data analysis.

Polyelectrolyte complexation was monitored by flowing varying concentrations of PEI over a PAZO-coated surface (1 mM). The resulting association curves and corresponding RU values are shown in Figure 2a. As expected, higher PEI concentrations produced faster observed rate constants (*k*_*obs*_) and larger equilibrium response values (RU_max_). Values of *k*_*obs*_ were obtained by fitting the association transients to a one-to-one binding model with single-phase exponential kinetics.

**Figure 2:**
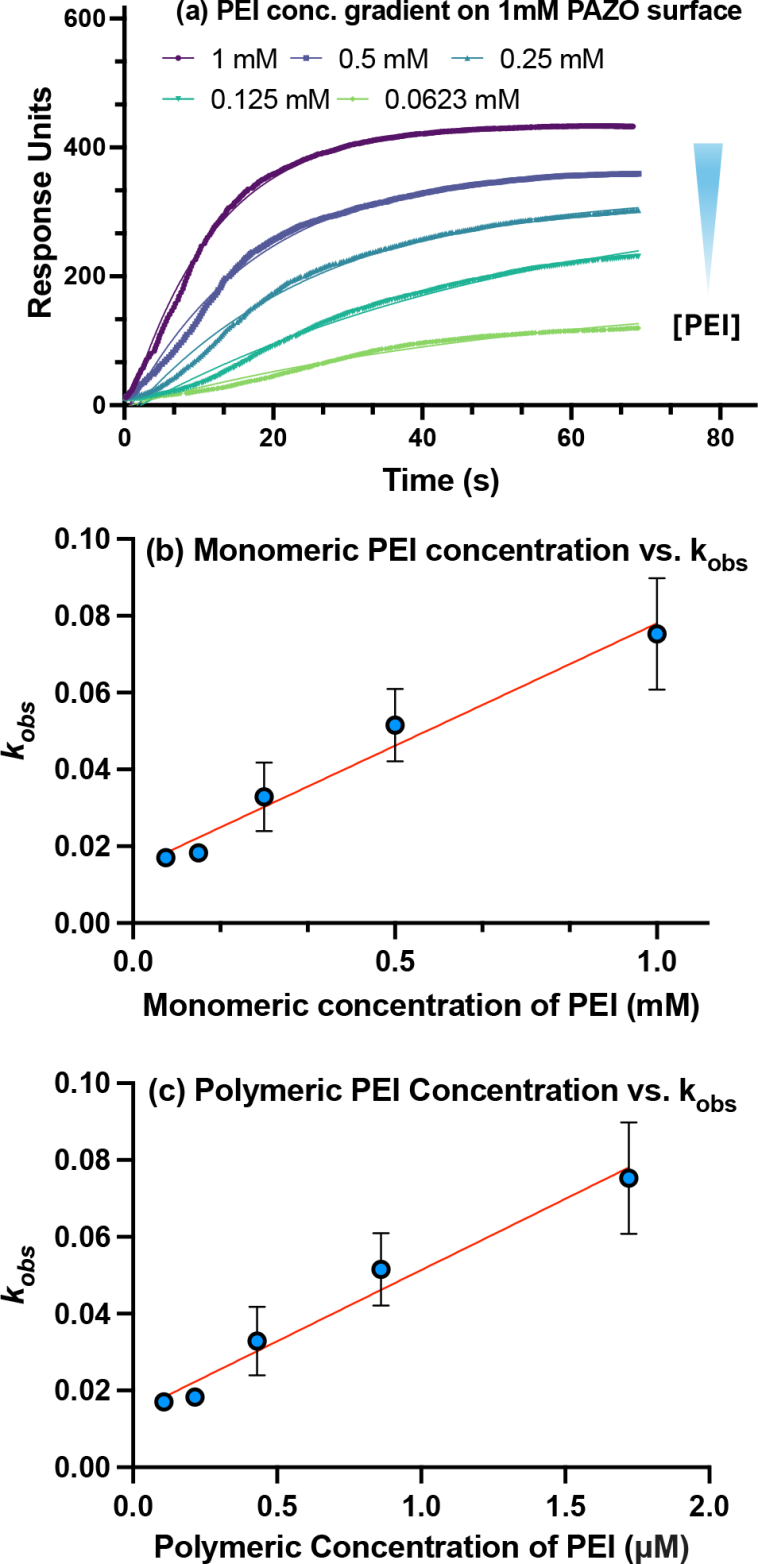
(a) LSPR association curves for PEI deposition onto a PAZO-coated surface (1 mM). At time zero, PEI solutions of the indicated monomeric concentrations were injected into the flow cell, and binding was monitored in real time until equilibrium was reached. (b) Observed rate constants (*k_obs_*) were extracted by fitting the association transients to a one-phase exponential binding model and plotted as a function of PEI monomeric concentration, in accordance with eq 6. (c) Plot of *k_obs_* versus polymeric concentration of PEI, obtained by converting monomeric to polymeric concentrations using the known number-average molecular weight (*M_n_*) of PEI and eq 8.

Observed rate constants (*k*_*obs*_) were determined for PEI at monomeric concentrations of 1, 0.5, 0.25, 0.125, and 0.0625 mM, yielding values of 0.07527 ± 0.01028, 0.05154 ± 0.00669, 0.03287 ± 0.00628, 0.0183 ± 0.00044, and 0.01701 ± 0.00076 s^–1^, respectively (mean ± SEM, *n* = 2). These results are plotted against the monomeric concentration in Figure 2b, in accordance with eq 6.

Because the binding process depends on polymer chains rather than individual repeat units, monomeric concentrations must be converted to polymeric concentrations. This conversion requires knowledge of the number-average molecular weight (*M*_*n*_) of PEI, which has been previously determined by SEC. However, it is important to note that SEC measurements of polyelectrolytes often carry significant uncertainty due to chain–column interactions and the reliance on calibration with neutral polymer standards. Thus, while SEC provides only an approximate value of *M*_*n*_, it enables the conversion of monomeric concentration [Monomeric] (based on the repeat-unit molar mass, *M*_*o*_) into polymeric concentration [Polymeric] (based on the chain molar mass, *M*_*n*_), as described by eq 8.

8 $$\left[\mathrm{Polymeric}\right]=\frac{\left[\mathrm{Monomeric}\right]M_{o}}{M_{n}}=\frac{\left[\mathrm{Monomeric}\right]}{DP}$$

DP in eq 8 is the degree of polymerization (defined as [*M*_*n*_/*M*_*o*_] and provides the average number of monomeric repeat units in the polymer.

After converting PEI concentrations (Table 2) from monomeric to polymeric units using *M*_*n*_, plots of polymeric concentration versus *k*_*obs*_ yielded the kinetic constants *k*_*on*_ and *k*_*off*_ (Figure 2c). This representation is more chemically meaningful than monomer-based values, as binding occurs at the level of entire polymer chains rather than individual repeat units. From the linear fit, *k*_*on*_ and *k*_*off*_ were determined to be 0.03705 ± 0.00986 μM^–1^ s^–1^ and 0.01430 ± 0.00875 s^–1^, respectively. The equilibrium dissociation constant, *K*_*D*_ = *k*_*off*_ /*k*_*on*_, was calculated to be 386 nM, consistent with a strong, electrostatically driven interaction between PEI and PAZO.

**Table 2:** Conversion of PEI Monomeric Concentrations to Polymeric Chain Concentrations Using Equation 8 ^a^

[Monomeric] (mM)	[Polymeric] (μM)	*k*_*obs*_ (s^–1^)
1.0000	1.7200	0.07527 ± 0.01028
0.0500	0.8600	0.05154 ± 0.00669
0.0250	0.4300	0.03287 ± 0.00628
0.0125	0.2150	0.01830 ± 0.00044
0.0625	0.1075	0.01701 ± 0.00076

a Monomeric concentrations were calculated based on the molar mass of the repeat unit (*M*_*o*_), while polymeric concentrations were obtained by normalizing to the number-average molecular weight (*M_n_*) of PEI. This conversion allows kinetic analysis to be performed in terms of polymer chains—the true binding entities—rather than individual repeat units.

Complexation kinetics were also measured for PAZO by flowing varying concentrations of the polyanion (calculated on a monomeric basis) over a PEI-coated surface (1 mM). The resulting association curves and corresponding RU values are shown in Figure 3a. As with PEI, higher PAZO concentrations produced faster observed rate constants (*k*_*obs*_) and larger equilibrium responses (RU_max_). Values of *k*_*obs*_ were obtained by fitting the association transients to a one-to-one binding model with single-phase exponential kinetics. Both single- and double-exponential fits were applied (Figure S1). While the double-exponential model yields a closer visual fit with smaller residuals, both models exhibit some systematic asymmetry in the residuals, which may reflect complex underlying kinetics such as multiphasic binding or conformational rearrangements, as is often observed in polyelectrolyte systems. Despite these deviations, all fits yielded high coefficients of determination (*R* ^2^ > 0.95), indicating that both models explain most of the variance in the data. Our primary goal in this analysis is to extract the observed rate constant *k*_*obs*_ for each PAZO concentration to enable downstream kinetic evaluation. The single-exponential model provides a more interpretable and consistent framework for this purpose. In contrast, the double-exponential model introduces additional parameters that lack clear mechanistic interpretation and are poorly constrained, particularly at lower concentrations. Although we cannot rule out the presence of more complex binding behavior, the single-exponential fit captures the dominant kinetic phase and offers a robust and meaningful estimation of *k*_*obs*_ values suitable for kinetic modeling and comparison.

**Figure 3:**
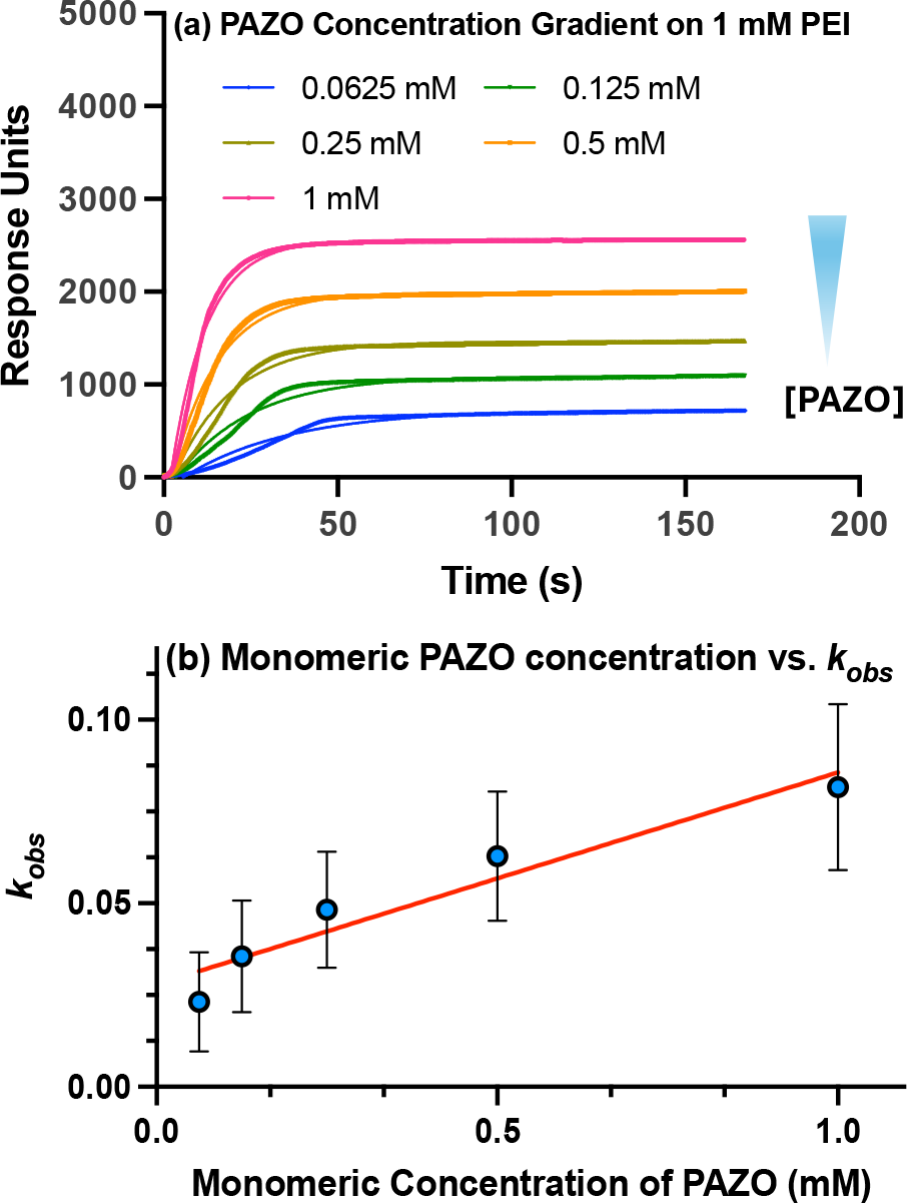
(a) LSPR association curves for PAZO deposition onto a PEI-coated surface (1 mM). At time zero, PAZO solutions of the indicated monomeric concentrations were injected into the flow cell, and binding was monitored in real time until equilibrium was reached. (b) Observed rate constants (*k*_*obs*_) were extracted by fitting the association transients to a one-phase exponential binding model and plotted as a function of PAZO monomeric concentration, in accordance with eq 6.

Figure 3b presents the dependence of *k*_*obs*_ on PAZO concentration, with values of 0.08162 ± 0.01599, 0.06280 ± 0.01245, 0.04830 ± 0.01123, 0.03560 ± 0.01076, and 0.02315 ± 2 0.00953 s^–1^ for concentrations of 1, 0.5, 0.25, 0.125, and 0.0625 mM, respectively (mean ± SEM, *n* = 2).

For PAZO, the polymeric concentration cannot be directly determined because its number-average molecular weight (*M*_*n*_) is unknown. By rearranging the expressions in eqs 6 and 8, the relationship can be reformulated to give eq 9:

9 $$k_{obs}=\frac{k_{on}\times \left[\mathrm{Monomeric}\right]}{DP}+k_{off}$$

In this expression DP = *M*_*n*_/*M*_*o*_ and represents the degree of polymerization of PAZO. Collecting terms gives

10 $$k_{obs}=\alpha \left[\mathrm{Monomeric}\right]+k_{off}$$

where

11 $$\alpha =\frac{k_{on}}{DP}=\frac{k_{on}\times M_{o}}{M_{n}}$$

The slope (α) of the plot in Figure 3b corresponds to the concentration-dependent term in the kinetic model. Because the fundamental association constant (*k*_*on*_) must be identical for both orientations of the interaction—PEI binding to PAZO or PAZO binding to PEI—the value of *k*_*on*_ obtained from the PEI analysis (Figure 2c) can be applied to the PAZO data to solve for its number-average molecular weight (*M*_*n*_). Using this approach, the *M*_*n*_ of PAZO was calculated to be 257,400 g mol^–1^. From this, the degree of polymerization (DP) was determined by dividing *M*_*n*_ by the repeat unit molar mass (*M*_*o*_), giving an average of 642 PAZO monomers per chain.

A key assumption in this analysis is that the intrinsic association rate constant (*k*_*on*_) is identical for both orientations of the interaction, whether PEI binds to PAZO or PAZO binds to PEI. This assumption is justified by the reciprocal nature of electrostatic attraction: the probability of encounter between a cationic repeat unit on PEI and an anionic repeat unit on PAZO does not depend on which polymer is immobilized, and which is in solution. In both experimental configurations, the fundamental elementary step is the same—local complexation between oppositely charged repeat units—so long as solution conditions such as ionic strength, temperature, flow rate, and surface coverage remain constant. Although surface immobilization can, in principle, influence local chain mobility or steric accessibility, it is not experimentally feasible to determine the intrinsic solution-phase association rate constant (*k*_*on*_) independently for both orientations of a polymer–polymer pair. All surface-sensitive kinetic methods inherently require one partner to be tethered, making direct experimental verification of perfect *k*_*on*_ symmetry impossible. Consequently, this proof-of-concept framework necessarily relies on the standard assumption, widely used in SPR/LSPR protein–protein studies and polyelectrolyte adsorption analyses, that the association-limited step is orientation-independent under conditions where long-range electrostatics dominate.^5,11,12,21,22,24,26–28^ In support of this assumption, both experimental orientations in our system (PEI associating with surface-bound PAZO and PAZO associating with surface-bound PEI) exhibited linear *k*_*obs*_–concentration relationships with comparable slopes and no signatures of diffusion-limited distortion. These observations indicate that the association process is governed primarily by long-range Coulombic attraction and remains effectively orientation-independent under the dilute, salt-free, extended-chain conditions used here. This assumption relies on identical experimental conditions like pH, temperature, and adsorption density which were controlled to our greatest ability. However, the dissociation rate constant (*k*_*off*_) may vary slightly between orientations due to differences in surface crowding or chain mobility, but such variations are expected to be minor relative to the strong electrostatic driving force that dominates the association process. Treating *k*_*on*_ as symmetric across both orientations therefore provides a valid basis for calculating the number-average molecular weight of PAZO from the kinetic data.

Finally, it should be noted that in systems with weaker electrostatic interactions, greater structural asymmetry, or significant conformational flexibility, deviations in *k*_*on*_ between orientations may arise, and the approach described here would need to be applied with caution. Also, this method may not extrapolate well to systems of extremely high or low molecular weights and must be independently investigated. Despite this limitation, the present study highlights how kinetic analysis via LSPR offers a powerful and calibration-free alternative to conventional SEC, enabling molecular weight determination of polyelectrolytes under conditions where traditional techniques fail. In summary, this work establishes LSPR-based kinetics as a practical and broadly adaptable strategy for characterizing charged polymers in salt-free environments.

## Conclusions and Future Directions

This work demonstrates a novel application of LSPR for the quantitative characterization of polyelectrolytes, introducing a kinetic framework that enables the determination of number-average molecular weight (*M*_*n*_) and degree of polymerization (DP) under salt-free conditions. Unlike traditional approaches such as SEC, which often require added salt, calibration against neutral polymer standards, or specialized instrumentation, the LSPR-based method provides a direct, calibration-free measurement derived from real-time surface binding kinetics. By monitoring the reciprocal electrostatic complexation of PAZO and PEI, and leveraging the symmetry of the association rate constant (*k*_*on*_) across experimental orientations, this study establishes a strategy for estimating molecular parameters that are otherwise difficult to obtain. The method expands the scope of LSPR beyond biomolecular sensing into polymer science, offering a powerful complement to conventional analytical techniques. Importantly, it highlights the broader utility of surface-sensitive kinetic measurements for probing intermolecular interactions of charged macromolecules. Because the approach is fast, adaptable, and does not rely on external calibration or perturbation of solution conditions, it provides a valuable new tool for polymer chemists, materials scientists, and surface engineers. Looking forward, this framework can be readily extended to other polyelectrolytes and biomolecular systems, underscoring its potential as a versatile platform for characterizing complex macromolecular interactions at interfaces. Overall, this study should be viewed as a proof-of-concept, demonstrating that LSPR-based kinetic measurements can provide a viable pathway to polyelectrolyte molecular weight determination, while leaving full validation and broader application to future investigations. We are in the process of performing additional studies on different polyelectrolytes, including a more detailed mass transport analyses to determine the degree of effect of diffusion control in polyelectrolyte multilayer self-assembly.

It should be emphasized that this work is deliberately limited in scope, employing a single-model polycation–polyanion pair (PEI and PAZO) to establish the feasibility of the approach. As such, the present results should be viewed as a methodological demonstration rather than a comprehensive validation across diverse systems. Future studies will be needed to test the robustness of the framework against polyelectrolytes of varying charge density, topology, and ionic environment, and to benchmark directly against conventional methods such as SEC or light scattering. Nevertheless, by clearly illustrating how surface-sensitive kinetic measurements can yield number-average molecular weight under salt-free conditions, this proof-of-concept study provides a foundation for broader applications in both synthetic polymer and biomolecular contexts.

## Supplementary Material



## Notes

Representative association sensograms fit with single- and double-exponential kinetic models, with corresponding residuals used to assess model adequacy (PDF)
