# Supplementary material for: An LSPR-Based Kinetic Framework for Polyelectrolyte Molecular Weight Determination: A Proof-of-Concept Study
Source: Anal Chem. 2026 Jan 21;98(4):2807–14. doi: 10.1021/acs.analchem.5c05449 (PMC12874201; doi:10.1021/acs.analchem.5c05449)
Supplement: Supplementary file 1 [file ac5c05449_si_001.pdf]

Supporting Information for

An LSPR-Based Kinetic Framework for Polyelectrolyte Molecular Weight Determination: A Proof-of-Concept Study

Ryan M. Mooney[1], Charles Brainin[1], and Malkiat S. Johal[1*]

[1] Department of Chemistry, Pomona College, Claremont, California 91711, United States

Table of Contents

Figure S1…………..………………………………………………………………………. S3

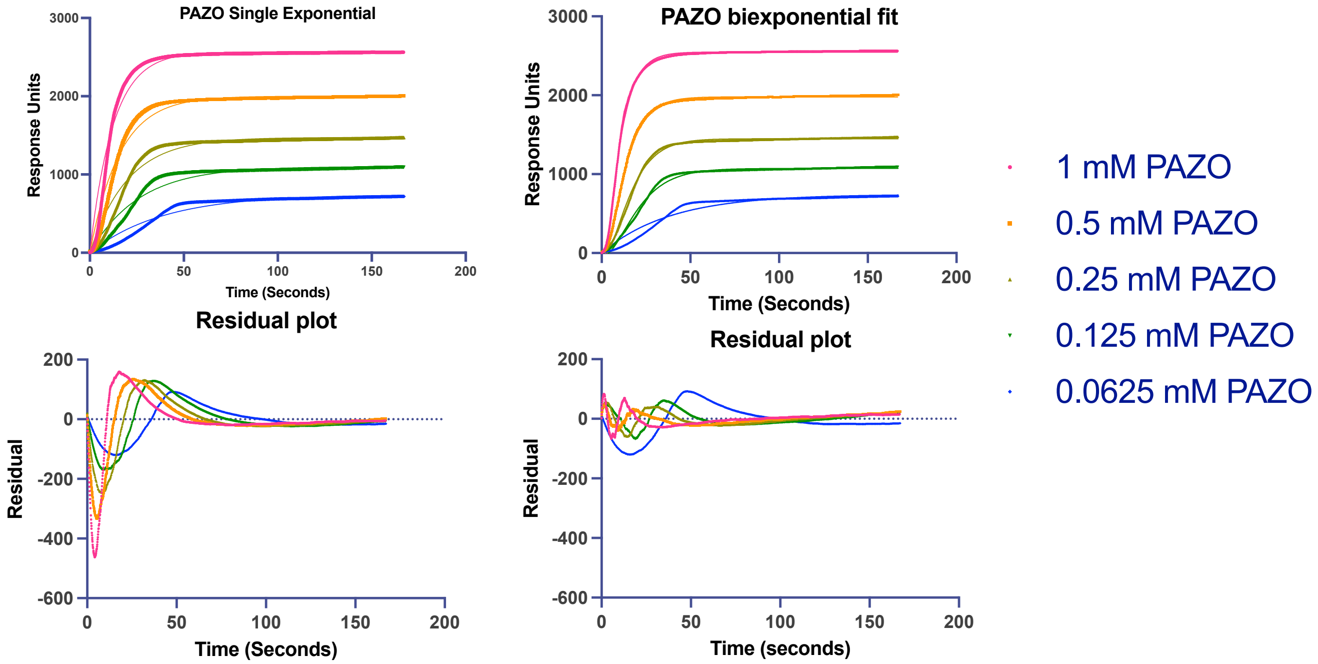

Figure S1. Kinetic model resolution. Representative association phase sensorgrams (top row) were fit using single exponential (left) and biexponential association models (right), with corresponding residuals shown below each fit. The residuals for the fits to the kinetic data displayed some systematic asymmetry. Fits to more complicated models did improve the residuals, but these models could be excluded due to experimental design of LSPR data analysis or nonconverging and unrealistic parameters. However, we cannot rule out a more complicated, multivalent model that may yield more robust k_{obs} values.
